# The association of painful and non-painful morbidities with frailty: a cross sectional analysis of a cohort of community dwelling older people in England

**DOI:** 10.1186/s12877-023-04602-w

**Published:** 2024-02-15

**Authors:** W. J. Chaplin, H. R. Lewis, S. M. Shahtaheri, B. S. Millar, D. F. McWilliams, J. R. F. Gladman, D. A. Walsh

**Affiliations:** 1 Academic Rheumatology, Injury, Recovery and Inflammation Sciences, University of Nottingham, Nottingham, England; 2 Pain Centre Versus Arthritis, University of Nottingham, Nottingham, England; 3 NIHR Biomedical Research Centre, University of Nottingham, Nottingham, England; 4 School of Medicine, University of Nottingham, Nottingham, England; 5 Centre for Rehabilitation & Ageing Research, Injury, Recovery and Inflammation Sciences, University of Nottingham, Nottingham, England; 6 Sherwood Forest Hospitals NHS Foundation Trust, Mansfield, Nottingham England; 7 Academic Rheumatology, Clinical Sciences Building, Nottingham City Hospital, Hucknall Road, Nottingham, NG5 1PB England

**Keywords:** Chronic pain, Frailty, Older people, Morbidities

## Abstract

**Introduction:**

The association between chronic pain and frailty might indicate that pain is an independent driver of frailty but might alternatively be explained by inclusion within frailty identification tools of morbidities that commonly lead to chronic pain. This research examines the extent to which the association of pain with frailty might be attributed to morbidities.

**Methods:**

A cross-sectional analysis of older people in a UK cohort with or at risk of musculoskeletal problems or frailty (Investigating Musculoskeletal Health and Wellbeing study), used multivariable logistic regression and Z-tests to assess the degrees of associations of pain (McGill Pain Rating Index), and painful and non-painful morbidity counts with frailty (modified FRAIL questionnaire).

**Results:**

Data were from 2,185 participants, 56% female, median age 73 (range 60 to 96) years. 430 (20%) participants were classified as frail. In a fully adjusted standardised model, pain (aOR 2.07 (95%CI 1.83 to 2.33) and ‘any’ morbidity aOR (1.74 (95%CI 1.54 to 1.97) were both significantly associated with frailty. When morbidity was subclassified as painful or non-painful, painful (aOR 1.48 (95%CI 1.30 to 1.68) and non-painful (aOR1.39 (95%CI 1.24 to 1.56)) morbidities each were associated with frailty, as also was pain (aOR 2.07 (95%CI 1.83 to 2.34, *p* < 0.001).

**Conclusions:**

Pain is associated with frailty, over and above any effect of painful and non-painful morbidities. This forms the justification for future research which focuses on pain management in the identification, prevention, and treatment of frailty.

**Supplementary Information:**

The online version contains supplementary material available at 10.1186/s12877-023-04602-w.

## Introduction

Frailty is a vulnerability state in older people that is characterised by a loss of homeostatic resilience as a consequence of multi-organ, age-associated decline [1]. Frailty is conceptualised using cumulative deficit models such as the Frailty Index [2]. Alternatively, frailty may be defined as a phenotype which groups clinical characteristics for example the Frailty Phenotype [3]. This research uses a hybrid frailty identification tool based on both models (FRAIL [4]). The prevalence of frailty in the UK is estimated at 15% of people over 65 years [5]. People with frailty have higher mortality, higher hospitalisation rates and are more disabled than those who are non-frail.

Frailty, whether classified according to cumulative deficit [2, 6–10] or phenotype models [3, 7, 11–13] or the hybrid FRAIL [4], is associated with chronic pain (pain unresolved after three months [14]). Musculoskeletal conditions are the most common causes of chronic pain affecting over a third of the UK population [15, 16]. An estimated 8.5 million people in the UK have osteoarthritis [15].

Frailty is associated also with multi-morbidity (two or more long-term health conditions [17]). In fact, multi-morbidity is an integral part of frailty identification tools based on the cumulative deficit model [2, 6–10] and FRAIL [4]. Accumulated deficits including those from morbidities represent multi-organ decline, and are associated with frailty classification [2, 18].

An association between chronic pain and frailty is identified both using the Fried phenotype model of frailty (which does not directly include morbidities) [3, 7, 11–13], and using the cumulative deficit models of frailty [2, 6–10]. This suggests that the association of pain with frailty is not purely a statistical phenomenon resulting from inclusion of morbidity counts in frailty identification tools. This raises the possibility that chronic pain might itself contribute to the frailty state. If so, chronic pain would be an additional variable that could be used in to identify, predict and measure frailty. Furthermore, attempts to ameliorate or manage chronic pain could potentially prevent or reverse frailty states. Current frailty interventions focus on other aspects such as exercise and nutrition [19].

We set out to examine the extent to which association of chronic pain with frailty might be attributed to morbidities.

## Methods

We performed cross-sectional analysis of data from participants recruited to the Investigating Musculoskeletal Health & Wellbeing (IMH&W) study, based in the East Midlands, UK (20).

### Participants and data sources

To participate in the IMH&W study participants were aged 18 years and over, and able to give informed consent and had completed baseline postal IMH&W questionnaires (n = 5,500). The eligible data extracted for this study in July 2020 included participants aged ≥ 60 years who completed all 5 items of the FRAIL questionnaire.

IMH&W recruited participants through multiple pathways, including inviting people with or at risk of musculoskeletal problems or frailty [20]. To enhance representation of participants with frailty in IMH&W, one recruitment pathway involved patients from General Practitioners (GP) registers with an electronic Frailty Index (eFI) score of ≥ 0.12 (threshold for mild frailty) [21]. The eFI comprises 36 deficits, consisting of morbidities, symptoms, activity/ mobility restrictions, social vulnerability, and care requirements [21].

This research was conducted under the ethics approval given for the IMH&W study by the Central London Research Ethics Committee (ref.18/LO/0870).

### Variables

The outcome variable is frailty, the predictors are morbidities and pain. Age, sex, and BMI were included as potential confounders. All variables are described below.

### Frailty

The IMH&W survey included the 5 self-report items comprising the FRAIL questionnaire [4]. Items were each scored as 0: not present, or 1: present. The 5 items were: **F**atigue (feeling tired all or most of the time in the last month); **R**esistance (difficulty ascending 10 steps without aid) and **A**mbulation (difficulty walking several hundred yards without aids) each scored as no or yes; **I**llnesses (≥ 5 from 11 specified conditions) and **L**oss of weight (> 5% during a year). The FRAIL items are used to classify people into non-frail (0 to 2 items) or frail (3 to 5 items). In the current study, to remove the overlap between FRAIL and morbidities, we modified FRAIL (“mFRAIL”). This omission of the illnesses (morbidity) item permitted examination of the contribution of morbidities to a frailty classification that approximates the phenotype model. Participants were classified using mFRAIL as non-frail (0 to 2 items) or frail (3 to 4 items).

The mFRAIL tool was assessed for validity using Receiver Operating Characteristic (ROC) curve analysis. The area under the ROC curve using the original FRAIL cutoff of 3/5 items = 0.997 and mFRAIL cut-off of 3/4 items was 0.986, *p* = 0.004.

### Morbidities

We generated an ‘any’ morbidity count variable (unweighted) which comprised the 11 conditions included in the illnesses item in the original FRAIL questionnaire (as above), plus 8 morbidities from the Charlson Comorbidity Index (CCI) [22] and 7 from the Functional Comorbidity Index (FCI) [23]. We ascertained these morbidities from:

1. A checklist of conditions prefaced with the question; ‘has a doctor told you that you have any of these conditions or problems,’ including the 11 FRAIL conditions and 7 other conditions (Additional Fig. 1).

2. Free text for “other conditions”, classified using criteria developed by consensus between WJC, DAW, and JRFG. Additional Table 1 shows the criteria for classifying “other” morbidities from free text. We performed this classification using an algorithm we developed using Excel and a series of logic and lookup commands. Two reviewers (WJC, SS) independently checked a sample of 100 participants and confirmed its reliability [ICC = 0.94 (95% CI 0.91 to 0.96), *p* < 0.001].

3. Participants’ self-reported medications by free text and/or prescriptions. WJC, DAW and JRFG reviewed and edited the list of participant medications to include only those medications specifically used for conditions included in our morbidities list, based on information in the British National Formulary (Additional Table 2). Morbidities were coded by algorithm, as above, according to self-reported use of these specific medications for each participant. For example, if a participant reported insulin, we inferred they had diabetes mellitus even if they had not listed it in the checklist or free text. Two reviewers (WJC, SS) independently checked a sample of 100 participants and confirmed its reliability [ICC = 0.98 (95% CI 0.97 to 0.99), *p* < 0.001].

We counted each morbidity once, drawing first from the morbidity checklist, secondly from free text and finally by inference from medication lists. Additional Table 3 shows the morbidities identified by this process.

We also classified the 26 morbidities as either ‘painful’ or ‘non-painful’ morbidities according to the International Association for the Study of Pain, Classification of Chronic Pain list of conditions for which pain management is routinely considered part of appropriate treatment [24].(Additional Table 3).

### Pain

In our primary analysis, pain was measured using the McGill Pain Rating Index [25, 26], to represent pain of any type or source. This instrument comprises 78 pain descriptors in 20 sets of words, which categorise pain into a common intensity dimension, in which a higher score indicates greater pain [25]. The descriptor rank value was based on the word position within each set. The Pain Rating Index equals the sum of the descriptor rank values, ranging from 1 to 78. Only one word may be ticked per set, if no words apply, a zero score was allocated. In confirmatory analysis we used a 0–10 Numerical Rating Scale (NRS) of joint pain intensity. Participants responded to the question: “over the past four weeks, how intense was your average pain or the average aching in your most bothersome joint, where 0 is “no pain”, and 10 is “pain as bad as could be”?”

### Other variables

Age (in years), sex (male/female), and body mass index class (BMI) (underweight/normal/pre-obese/obese) were included in all multivariable analyses. Weight (kg), and height (m) were from questionnaire self-report, from which BMI (kg/m^2^) was calculated. BMI was then classified using WHO categories [27]; obese sub-categories were collapsed into a single ‘obese’ category of BMI > 30. BMI was treated as categorical because those regarded as either underweight or obese might be less healthy than those with normal BMI.

### Statistical analysis

Data were summarised using medians and ranges for non-normally distributed variables, and n (%) for dichotomous variables. Normality was assessed graphically using histograms and statistically using the Shapiro-Wilk test. None of the assessed variables were found to be normally distributed. Differences between groups were evaluated using Mann-Whitney U tests for continuous variables, and Chi-squared test for categorical variables. Correlations were assessed using Spearman rho and bootstrapped (10,000) to derive confidence intervals. Cases with any missing data were excluded in regression analysis.

We validated the mFRAIL threshold by exploring the internal structure of FRAIL using Cronbach’s alpha and Receiver Operator Curve (ROC) analysis of mFRAIL score against original FRAIL classification.

Multivariable logistic regression analyses were used to examine the extent to which association of chronic pain with frailty can be attributed to morbidities. Change was assessed by comparing the association of chronic pain with frailty alongside when ‘any’ morbidity count was added to the model. Standardized coefficients permitted comparison of variables with different scales. They represent the change in the dependent variable’s standard deviation associated with a one-standard-deviation increase in the predictor variable.

To examine the extent to which association of morbidities with frailty can be attributed to pain, we used multivariable logistic regression analyses. We investigated associations between painful and non-painful morbidity count with frailty.

Age, sex, and BMI class were selected *a priori*, other co-variables were included if p < 0.05 in prior bivariate analysis.

Categorical variables were classified as a binary outcome (absent/present) in all logistic regression models. Coefficients were standardised by generating z scores ((value-mean)/ standard deviation)) to permit a direct comparison of the magnitude and impact of different variables on the dependent variable. Z-tests were used to compare the strengths of regression coefficients or the changes between coefficients in separate models [28], where Z values ≥ ± 2 were interpreted as significantly different coefficients.

Statistical analyses were undertaken using STATA SE v16 (StataCorp LLC), using p ≤ 0.05 to indicate statistical significance.

## Results

There were 5,550 baseline data records checked for eligibility, with 2,185 participants whose data met the eligibility criteria for this study. Their characteristics are shown in Table 1. Median age was 73 (range 60 to 96) years, and 1,202 (55%) were female. FRAIL and mFRAIL (FRAIL with illnesses excluded) classified respectively 430 (20%) and 418 (19%) as frail. Use of mFRAIL led to a re-classification of only 12/430 (3%) participants classified by FRAIL. The median (IQR) Pain Rating Index was 13 (7 to 22). Data missingness was: Pain Rating Index n = 233 (11%), sex n = 1, and BMI n = 29 (1%), with no missing data for frailty, age, or morbidity counts. A flow diagram with details of missing data which were excluded from analysis are shown in Additional Fig. 2.

**Table 1:** Participant characteristics at baseline

Variable	All participants *N* = 2,185	≥ 1 painful morbidities	≥ 1 non-painful morbidities
Sex:			
Male, *n* (%)	982 (45)	833 (44)	677 (45)
Female, *n* (%)	1,202 (55)	1,056 (56)	818 (55)
Age (years), median (range)	73 (60 to 96)	73 (60 to 96)	73 (60 to 96)
Ethnicity:			
White, *n* (%)	2,152 (99)	1,865 (98)	1,468 (98)
Non-white	13 (1)	23 (1)	28 (2)
Socioeconomic Status, median (IQR)			
Indices of Multiple Deprivation	8 (5–9)	8 (5–9)	8 (5–9)
BMI Classes^#^, *n* (%)			
Underweight	30 (1)	27 (1)	20 (1)
Normal	653 (32)	540 (29)	417 (28)
Pre-obese	831 (39)	723 (39)	552 (38)
Obese	642 (30)	573 (31)	482 (33)
FRAIL classification			
Frail	430 (20)	414 (22)	343 (23)
Non-frail	1,755 (80)	1,476 (78)	1,153 (77)
Pain Rating Index (1–78)			
median (IQR)	13 (7–22)	14 (7–22)	14 (7–22)
FRAIL illness item (≥ 5/11), *n* (%)	58 (2.7)	58 (2.7)	58 (2.7)
Morbidity count			
All, median (range)	3 (0 to 12)	3 (2–4)	3 (2–4)
Painful, median (range)	2 (0 to 8)	2 (1–3)	2 (1–3)
Non-painful, median (range)	1 (0 to 5)	1 (0–1)	2 (1–2)
Recruitment Route *n* (%)			
GP	1,972 (90)	1,697 (90)	1,372 (92)
Previous studies	191 (9)	173 (9)	110 (7)
Other	22 (1)	18 (1)	12 (1)

Abbreviations: SD – standard deviation; BMI – body mass index; FRAIL (unmodified);

Number of observations for each variable vary; 2,185 relates to complete FRAIL, and age data.

^#^WHO classification for BMI (kg/m2), underweight < 18.5, normal 18.5–24.9, pre-obese 25-29.9 and > 30 obese.

Note: the ≥ 1 painful and non-painful categories are for descriptive purposes. Participants may have both a painful and non-painful morbidity.

### Morbidities

Participants reported median (range) 3 (0 to 12) ‘any’ morbidities, 2 (0 to 8) painful morbidities, and 1 (0 to 5) non-painful morbidities. Only 96 (4%) participants reported no morbidities, and 1,297 (59%) participants had at least one painful plus one non-painful morbidity. The frequencies of morbidity counts are shown in Table 2. The most frequently reported painful and non-painful morbidities were arthritis 1,477 (68%) and hypertension 856 (39%), respectively. Higher ‘any’ morbidity count was associated with being female, pre-obese or obese. Higher painful morbidity count was associated with being female, older, or obese. Higher non-painful morbidity count was associated with obesity (Additional Table 4).

**Table 2:** IMH&W morbidity frequency by painful/non-painful classification (N = 2185)

Painful Morbidity	*n*, (%)	Painful / Non-painful
Arthritis	1,477 (68)	Painful
Hypertension	856 (39)	Non-painful
Degenerative disc disease	791 (36)	Painful
Upper gastro-intestinal	684 (31)	Painful
Asthma	413 (19)	Non-painful
Diabetes without complications	386 (18)	Non-painful
Osteoporosis	247 (11)	Painful
Cancer	237 (11)	Painful
Angina	228 (10)	Painful
Lung disease	185 (8)	Non-painful
Myocardial Infarction	150 (7)	Painful
Stroke	145 (7)	Non-painful
Kidney disease	124 (6)	Non-painful
Depression	104 (5)	Painful
Visual impairment	91 (4)	Non-painful
Chronic Heart Failure	63 (3)	Painful
Neurological	56 (3)	Non-painful
Anxiety	40 (2)	Non-painful
Lower Gastro-intestinal	29 (1)	Painful
Diabetes with complications	12 (0.5)	Painful
Hearing impairment	12 (0.5)	Non-painful
Dementia	9 (0.4)	Non-painful
Liver disease	7 (0.3)	Painful
Peripheral vascular disease	6 (0.3)	Painful
AIDS	1 (< 0.1)	Non-painful
Hemiplegia	1 (< 0.1)	Non-painful

Abbreviations: AIDS – Acquired immune deficiency syndrome. Items in bold are included in FRAIL.

Diabetes is classified as painful if the participant reports complications such as neuropathy (Charcot foot), or retinopathy.

Note this classification uses IASP Chronic Condition classification of conditions in which pain management is routinely considered as part of the treatment regime. In bivariate analyses, Pain Rating Index was positively correlated with the count of ‘any’ morbidities (r_s_= 0.24, 95% CI 0.19 to 0.28), *p* < 0.001). Painful morbidity counts were positively correlated with non-painful morbidity counts (r_s_= 0.10, 95% CI 0.06 to 0.15, *p* < 0.001). Pain rating index was more strongly correlated with painful morbidity count (r_s_= 0.26, 95% CI 0.22 to 0.31, *p* < 0.001) than with non-painful morbidity count (r_s_= 0.07 95% CI 0.02 to 0.11, *p* = 0.003).

### Bivariate associations of frailty with pain, morbidity, and covariates

Frailty (mFRAIL) was associated with pain, morbidity counts and co-variates.

In those classified as frail, the median Pain Rating Index was 22 (IQR 13 to 33) compared to 11 (IQR 6 to 19) in those who were non-frail. Pain Rating Index was associated with mFRAIL frailty classification (OR 2.23, 95% CI 2.00 to 2.50, *p* < 0.001).

‘Any’ (OR 2.04, 95% CI 1.83 to 2.28, *p* < 0.001), painful (OR 1.89, 95% CI 1.69, 2.10, *p* < 0.001) and non-painful (OR 1.50, 95% CI 1.36 to 1.67, *p* < 0.001) morbidity counts were each associated with mFRAIL frailty classification.

Age (OR 1.02, 95% CI 1.00 to 1.03, *p* = 0.045), female sex (OR 1.91, 95% CI 1.52 to 2.39, *p* < 0.001), and BMI class (underweight OR 3.21 95% CI 1.42 to 7.25, *p* = 0.005, pre-obese OR 1.50 95% CI 1.11 to 2.03, *p* = 0.008, obese OR 2.96 95% CI 2.21 to 3.97, *p* < 0.001) also each was associated with mFRAIL frailty classification.

### The extent to which association of chronic pain with frailty can be attributed to morbidities

In multivariable analysis, higher pain was associated with mFRAIL frailty classification (aOR 2.21 (95% CI 1.96 to 2.49), *p* < 0.001, when adjusted for age, sex, and BMI class (Table 3). When ‘any’ morbidity count was added to the model there was a non-significant (Z = 0.76) reduction in the contribution of pain to frailty classification (aOR 2.07 (95% CI 1.83 to 2.33), *p* < 0.001). When instead of ‘any’ morbidity, painful (aOR 1.48 (95% CI 1.30 to 1.68)) and non-painful (aOR 1.39 (95% CI 1.24 to 1.56)) morbidity counts were together included in the model, the contribution of pain to frailty classification was similar (aOR 2.07 (95% CI 1.83 to 2.34), *p* < 0.001), Z=-0.002) (Table 3).

**Table 3:** Associations of pain and other characteristics with frailty

Factor	Interval/category	Model			
		**Pain**		**Pain & ‘any’ morbidity count**	**Pain, painful and non-painful morbidity count**
Chronic Pain	Standardised Pain Rating Index	2.21 (1.96, 2.49), p < 0.001		2.07 (1.83, 2.33), *p* < 0.001	2.07 (1.83, 2.34), *p* < 0.001
‘Any’ morbidity	Standardised count	Not included		1.74 (1.54, 1.97), *p* < 0.001	Not included
Painful morbidity	Standardised count	Not included		Not included	1.48 (1.30,1.68), *p* < 0.001
Non-painful morbidity	Standardised count	Not included		Not included	1.39 (1.24, 1.56), *p* < 0.001
Sex	Male	Ref		Ref	Ref
	Female	1.56 (1.21, 2.00), *p* = 0.001		1.55 (1.20, 2.01), *p* = 0.001	1.56 (1.21, 2.02), *p* = 0.001
Age	Years	1.05 (1.03, 1.07), *p* < 0.001		1.04 (1.02, 1.06), *p* < 0.001	1.04 (1.02, 1.06), *p* < 0.001
BMI Class^#^	Underweight	2.54 (1.02, 6.37), *p* = 0.046		2.80 (1.10, 7.12), *p* = 0.031	2.82 (1.11, 7.18), *p* = 0.030
	Normal	Ref		Ref	Ref
	Pre-obese	1.43 (1.03, 1.98), *p* = 0.033		1.42 (1.01, 1.99), *p* = 0.041	1.42 (1.02, 1.99), *p* = 0.040
	Obese	2.37 (1.71, 3.29), *p* < 0.001		2.25 (1.60, 3.14), *p* < 0.001	2.24 (1.60, 3.13), *p* < 0.001
Pseudo r^2^		0.1412		0.1832	0.1822

The outcome in each multivariable model was frailty classification (binary), defined as mFRAIL score > 2. Data are aOR (95%CI) from n = 1925 participants. Standardized coefficients represent the change in the dependent variable’s standard deviation associated with a one-standard-deviation increase in the predictor variable, they permit comparison of variable with different scales. The pain model is frailty adjusted for pain, age, sex, and BMI class. The pain and ‘any’ morbidity count model is adjusted for pain, ‘any’ morbidity count, age, sex, and BMI class. The pain & painful morbidity count model is adjusted for pain, painful and non-painful morbidity count, age, sex, and BMI class. Abbreviations: BMI – Body Mass Index; aOR –adjusted odds ratio; CI – 95% confidence intervals; Ref - reference group. ^#^WHO classification for BMI (kg/m2), underweight < 18.5, normal 18.5–24.9, pre-obese 25-29.9 and > 30 obese.

Similar findings were found in secondary analyses using NRS joint pain scores instead of the Pain Rating Index: pain (aOR 3.32, (95%CI2.79, 3.95), p < 0.001), painful (aOR 1.37, (95%CI 1.20, 1.56), p < 0.001) and non-painful (aOR 1.39, (95%CI 1.24, 1.58), p < 0.001) morbidities were significantly associated with mFRAIL frailty classification (Additional Table 5).

### The extent to which association of morbidities with frailty can be attributed to pain

Higher ‘any’ morbidity count (aOR 1.98 (95% CI 1.77 to 2.23, *p* < 0.001)); higher painful (aOR 1.84, 95% CI 1.65 to 2.06, *p* < 0.001) and higher non-painful (aOR 1.49, 95% CI 1.34 to 1.66, *p* < 0.001) morbidity counts were associated with mFRAIL frailty classification in separate multivariable regression models, each of which included age, sex, and BMI class as covariates (Table 4). Both painful and non-painful morbidity counts remained significantly associated with mFRAIL frailty classification when they were included in a single age-, sex-, and BMI- adjusted model (painful morbidity count aOR 1.67, (95% CI 1.49 to 1.88, *p* < 0.001, non-painful morbidity count aOR 1.38, (95% CI 1.24 to 1.55, *p* < 0.001). When Pain Rating Index was added to this model, painful and non-painful morbidity counts remained significantly associated with mFRAIL frailty classification (Table 3), although the effect of painful morbidity count was slightly reduced and became similar to that of non-painful morbidities.

**Table 4:** Associations of morbidity counts and other characteristics with frailty

Factor	Interval/category	Model		
		**Any morbidities**	**Painful morbidities**	**Non-painful morbidities**
Any morbidity	Standardised count	1.98 (1.77, 2.23), *p* < 0.001	Not included	Not included
Painful morbidity	Standardised count	Not included	1.84 (1.65, 2.06), *p* < 0.001	Not included
Non-painful morbidity	Standardised count	Not included	Not included	1.49 (1.34, 1.66), *p* < 0.001
Sex	Male	Ref	Ref	Ref
	Female	1.96 (1.54, 2.49) p < 0.001	1.89 (1.49, 2.40), *p* < 0.001	2.03 (1.61, 2.57), *p* < 0.001
Age	Years	1.02 (1.01, 1.04), *p* = 0.005	1.02 (1.01, 1.04), *p* = 0.005	1.03 (1.01, 1.04), *p* = 0.001
BMI Class^#^	Underweight	2.93 (1.24, 6.92), *p* = 0.014	2.79 (1.19, 6.53), *p* = 0.018	3.06 (1.33, 7.04), *p* = 0.008
	Normal	Ref	Ref	Ref
	Pre-obese	1.60 (1.16, 2.19), *p* = 0.004	1.63 (1.19, 2.22), *p* = 0.002	1.66 (1.22, 2.26), *p* = 0.001
	Obese	2.99 (2.18, 4.10), *p* < 0.001	3.21 (2.35, 4.39), *p* < 0.001	3.18 (2.34, 4.32), *p* < 0.001
Pseudo r^2^		0.1225	0.1076	0.0763

The outcome measure was frailty classification (binary), defined as mFRAIL score > 2. Data are aOR (95%CI) from *n* = 2155 participants. Standardized coefficients represent the change in the dependent variable’s standard deviation associated with a one-standard-deviation increase in the predictor variable, they permit comparison of variable with different scales. The first multivariable model is frailty adjusted for ‘any’ morbidity count, age, sex, and BMI class, the second is adjusted for painful morbidity count, age, sex, and BMI class and the third is adjusted for non-painful morbidity count, age, sex and BMI class. Abbreviations: BMI – Body Mass Index; aOR –adjusted odds ratio; CI – 95% confidence intervals; Ref - reference group. ^#^WHO classification for BMI (kg/m2), underweight < 18.5, normal 18.5–24.9, pre-obese 25-29.9 and > 30 obese.

## Discussion

We found that pain, painful and non-painful morbidity counts were all associated with frailty when included in a single multivariable model. Inclusion of morbidities in any model did not substantially reduce the relationship between chronic pain and frailty, indicating that this relationship is unlikely to be explained entirely by morbidities.

Our findings confirm and help to elucidate the previously demonstrated association between pain and frailty [6, 7, 10–12, 29]. Pain is associated with morbidities, but multimorbidity alone does not explain the effect of pain on frailty classification. It might be that pain or the process through which pain is experienced leads to frailty, or that loss of resilience inherent in the frailty state predisposes people to chronic pain. Others have described the relationship between morbidities and frailty [1, 18, 30–33], however, they have not explored this in the context of the relationship between pain and frailty. This research confirms the relationship between morbidities and frailty, and, to our knowledge, this is the first study to explore morbidity classification by pain.

A clinical implication of our findings, given that chronic pain is highly prevalent [15, 16], is that effective pain management might have great potential to prevent or reduce frailty in the community. Another implication, for those who study frailty, is that chronic pain might be a factor that could be used in the identification, measurement, and prediction of frailty.

Our study has both strengths and limitations. Although different results might have been obtained in different populations, our sample was representative of the IMH&W cohort, was large and had a high prevalence of painful and non-painful morbidities, pain, and frailty, enabling detailed exploration of these relationships. Our sample had an approximately equal male to female distribution and included people from a range of socioeconomic backgrounds. However, IMH&W itself selectively recruited people with or at risk of frailty or musculoskeletal problems and displayed little ethnic diversity.

Different results might have been obtained using different frailty classification tools and future work should include other frailty classification tools to confirm our findings. IMH&W was a postal questionnaire survey and so it was not possible for us to use in-person measurement of gait speed and grip strength to classify frailty phenotype [3]. We modified the FRAIL classification criteria by removal of the illness item in order to investigate the role of morbidities in the relationship between pain and frailty. However, mFRAIL and FRAIL only classified 12 (3%) participants differently, suggesting that mFRAIL and FRAIL have similar validity for frailty classification. FRAIL has previously been shown to be a valid tool for frailty classification [4, 34], which performs comparably with other frailty tools [35, 36]. Our findings, however, suggest that mFRAIL and FRAIL might not fully describe frailty, and other frailty classifications might give different results.

We obtained similar findings using two different pain measurement tools (Pain Rating Index and NRS). However, pain is a complex, multidimensional symptom and other pain measurement tools could give different results. It remains possible that aspects of pain (e.g., lower limb joint pain) result in an overclassification of frailty due to the inclusion in frailty classification tools of physical activity.

A strength of our study is that we found an association between pain and frailty, even after using an extensive list of morbidities to measure morbidity counts. We acknowledge the imprecision of classifying morbidities as either painful or non-painful using IASP criteria for conditions where pain management should be considered. Pain may be reported in conditions such as stroke that were classified as non-painful. Future research might assess effects of differentially weighting specific morbidities, although our findings suggest that weighting painful and non-painful morbidities differently would be unlikely to substantially affect frailty classification, nor the association of pain with frailty.

We acknowledge the risk of residual confounding due to the inclusion of inter-correlated variables in our multivariable models.

Longitudinal and interventional study designs are required to determine causality of the relationships we observed between pain, morbidities, and frailty. Future research should explore mechanisms by which pain might lead to frailty, for example by reducing physical activity, impairing appetite and nutrition, or through neuro-endocrine dysregulation. Randomised controlled trials would be required to test whether interventions that improve pain (even if not directly addressing underlying morbidities) can prevent or reverse frailty. A range of interventions that can reduce chronic pain (e.g., psychological, pharmacological, surgical, physical) might be explored in populations with or at risk of frailty, aiming not only to reduce pain, but also to facilitate transition to a non-frail state, or prevent transition into frailty.

In conclusion, chronic pain and multi-morbidity are both associated with frailty. The relationship of pain with frailty cannot be explained by morbidities, and the relationship between morbidities and frailty is not explained solely by pain. Further research is required to understand the complex relationship between pain and frailty. Interventions to mitigate the effect of chronic pain upon frailty should not be focussed solely upon treating underlying morbidities, but also manage chronic pain irrespective of its aetiology.

### Electronic supplementary material

Below is the link to the electronic supplementary material.

Supplementary Material 1

Supplementary Material 2

## Data Availability

The data that support the findings of this study are available from the authors upon reasonable request and with permission of the University of Nottingham. Enquires should be sent to David.Walsh@nottingham.ac.uk.
