# Supplementary material for: The association of painful and non-painful morbidities with frailty: a cross sectional analysis of a cohort of community dwelling older people in England
Source: BMC Geriatr. 2024 Feb 15;24:158. doi: 10.1186/s12877-023-04602-w (PMC10870594; doi:10.1186/s12877-023-04602-w)
Supplement: Supplementary file 1 — Supplementary Material 1 [file 12877_2023_4602_MOESM1_ESM.pdf]

The association of painful and non-painful morbidities with frailty: a cross sectional analysis of a cohort of community dwelling older people.

Additional Figures

Additional Figure 1. IMH&W Questionnaire medical condition checklist.

Additional Figure 2. IMH&W Flow Diagram with missing data details.

Additional Tables

Additional Table 1. Illness criteria for classification of morbidity free text.

Additional Table 2. Medications indicating specific illnesses.

Additional Table 3. Morbidity count by checklist and augmentation (n=6,342)

Additional Table 4. Bivariate associations between morbidity count and covariables.

Additional Table 5. Associations of frailty with standardised NRS pain and other characteristics.

- **Additional Figure 1. IMH&W Questionnaire medical condition checklist.**

Has a doctor told you that you have any of these medical conditions or problems?
If yes, please place a tick in the boxes provided.

- ☐ Angina
- ☐ Arthritis
- ☐ Asthma
- ☐ Back or spine problems
- ☐ Cancer (not minor skin cancers)
- ☐ Dementia
- ☐ Diabetes mellitus
- ☐ Fibromyalgia
- ☐ Gout
- ☐ Heart attack
- ☐ Heart failure
- ☐ Hypertension
- ☐ Kidney disease
- ☐ Lung disease
- ☐ Osteoarthritis
- ☐ Osteoporosis
- ☐ Rheumatoid arthritis
- ☐ Stroke

Others (please specify any conditions not listed above)

Medicines including pain killers
Please write down the names of any medications, including any pain killers, that you use. They can be prescriptions or bought over the counter.

Names of medications

- **Additional Figure 2. IMH&W Flow Diagram with missing data details.**

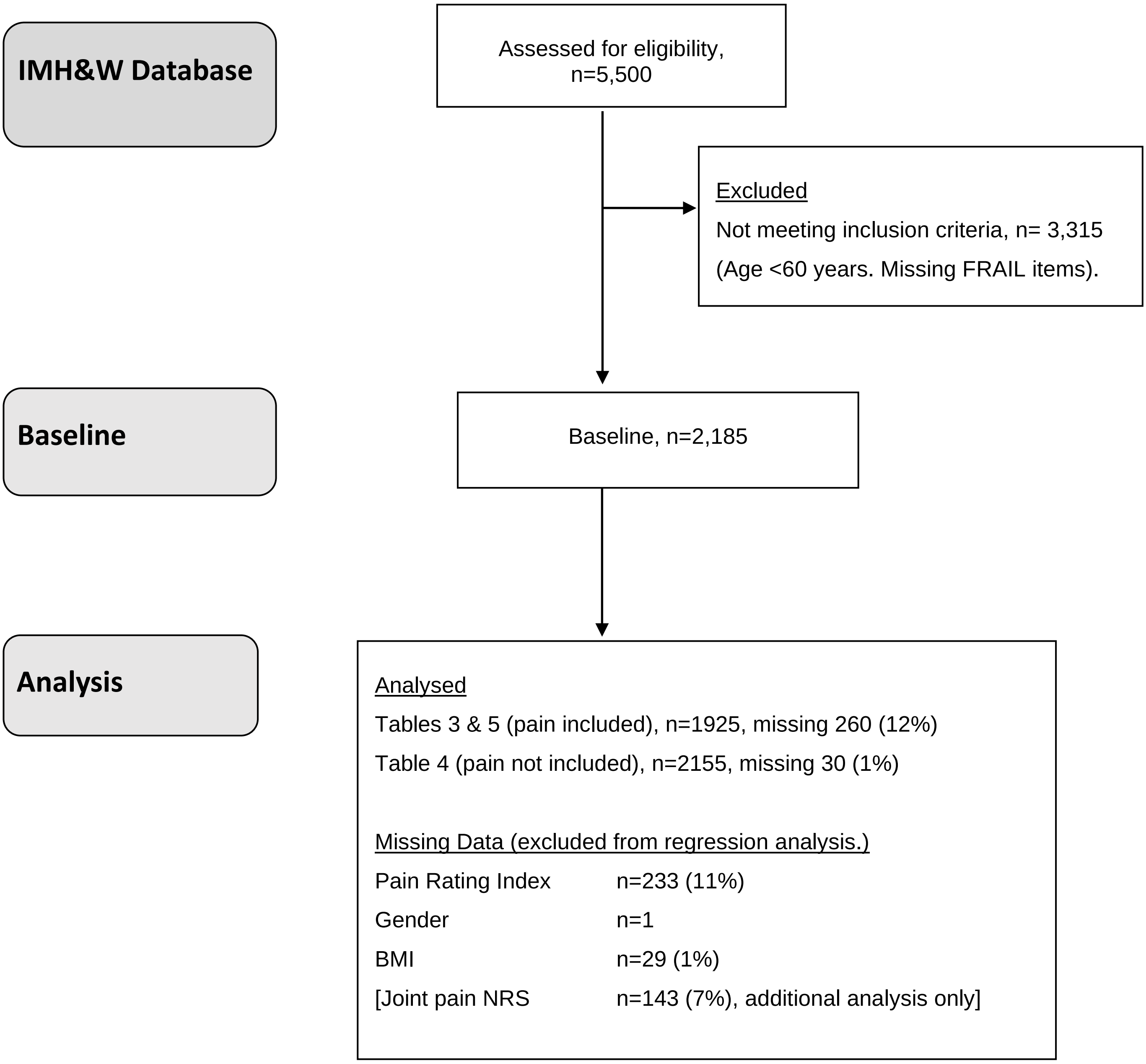

- **Additional Table 1. Illness criteria for classification of morbidity free text.**

**Illness condition**	**Illness inclusion criteria**
Diabetes	Include type 1 or 2 diabetes on medication; exclude if pre-diabetic or controlled by diet alone
Cerebrovascular disease	Include stroke or TIA; cavernous angiomas multiple brain, brain aneurysm, brain haemorrhage.
Hypertension	Hypertension or high blood pressure
Angina	Angina also include those who had coronary artery bypass graft, and those initially admitted with unstable angina. Include those who report insertion of stents and any heart bypass.
Congestive heart failure	Includes heart failure and exertional or paroxysmal nocturnal dyspnoea.
Myocardial infarction	Include if any heart attack old or new, includes cardiac arrest.
Osteoarthritis, gout	osteoarthritis and gout and arthritis
RA + connective tissue disease	Include all inflammatory arthritis, including ankylosing spondylitis, psoriatic arthritis, RA, and inflammatory systemic conditions such as polymyalgia rheumatica, systemic lupus erythematosus, lupus, polymyositis, seronegative arthritis.
Asthma	Include any form of possible reversible airways obstruction, whether or not there is additional fixed obstruction or other lung pulmonary deficits this means that a patient reporting to have only COPD who is prescribed one of these will also be classified as having asthma
Lung disease	Include any non-reversible-airway limitation. Chronic obstructive pulmonary disease (COPD), acquired respiratory distress syndrome (ARDS), emphysema, pulmonary embolism, and thromboembolic pulmonary hypertension or permanent lung impairment such as resection.
Renal disease	Include patients on dialysis, those who had a transplant, and those with uraemia.
Cancer	Include all cancers, tumours or mention of chemotherapy/ radiotherapy or other cancer treatment regardless of when it occurred. Exclude if minor skin cancer.

Note: Osteoarthritis and Rheumatoid arthritis categories were collapsed into one called arthritis.

- **Additional Table 2. Medications indicating specific illnesses.**

- **Angina**

Glyceryl trinitrate:
1-glyceryl nasal spray
2-gtn spray (mouth spray)
3-gtn tablets
4-nitromin spray
5-nitrolingual pump spray
Ranolazine
Nicorandil
Isorbide mononitrate
Isorbide dinitrate
Tardisc xl
Monomil xl
Isotard xl

- **Asthma**

Drugs in the class selective beta2-agonists (short-acting)
Salbutamol:
Salamol easi-breathe cfc-free inhaler Ventolin 100micrograms/dose evohaler
Drugs in the class inhaled corticosteroids:
Beclometasone dipropionate
Qvar easi-breathe inhaler
Clenil modulite inhaler
Budesonide with formoterol
Formoterol fumate
Symbicort 100/6 turbohaler
Beclometason with formoterol
Fostair nexthaler® 200/6
Fluticasone with salmeterol
Airflusal
Seretide evohaler
Beclomethasone dipropionate
Becotide 50 inhaler
Budesonide
Pulmicort turbohaler
Fluticasone with salmeterol
Salmeterol
Seretide evohaler
Seretide 250 accuhaler
Sirdupla inhaler
Terbutaline sulfate

Others:
Uniphyllin continus
Tiotropium
Aclidinium bromide with formoterol

Aclidinium bromide
Glycopyrronium with indacaterol
Nucala inhaler
Mepolizumab
Trimbow inhaler

- **Osteoarthritis, gout**

Colchicine
Alluprinol
Febuxostat

- **RA + connective tissue disease***

*Put in a conditional argument (rituximab if not lymphoma), all anti-tnfs if not ulcerative colitis/ Crohn’s

Drugs in the class tumour necrosis factor alpha (tnf-a) inhibitors:
Etanercept
Benepalil solution for injection pre-filled syringes
Enbrel solution for injection pre-filled myclic pens
Erelzsolution for injection pre-filled syringes

Drugs in the class disease-modifying anti-rheumatic:
Leflunomide tablets
Drugs in the class t-cell activation inhibitors:
Abatacept
Orencia solution for injection pre-filled syringes

Drugs in the class interleukin inhibitors:
Tocilizumab
Adalimumab
Benepali
Betamethasonevalerat
Hycophenalate
Hydroxychloroquine
Levo humira
Metoject
Nivestim
Methrotexate
Sulphasalazine
Leflunominde

All anti-tnfs (unless psoriasis or inflammatory bowel disease, rituximab, tocilizumab)

Infliximab
Cyclophosphamide

- **Diabetes**

Alogliptin
Basaglar insulin
Bolamyn SR
Dapagliflozin
Dulaglutide
Empagliflozin
Forxiga
Gliclazide
Gliclazide
Glimepiride
Glucophage
Humulin
Insulin
Januvia
Jardiance
Levemir insulin
Levo linagliptin
Linagliptin
Liraglutide
Metabet
Metformin
Novomix
Pioglitazone
Sitagliptin
Sukkarto
Synjardy
Toujeo
Trajenta
Victoza
Vipidia
Zicron

- **Hypertension**

Calcium-channel blockers:
Lacidipine
Lercanidipine hydrochloride
Securon SR tablets
Drugs in the class non-diuretic thiazide:
Indapamide
Drugs in the class antihypertensives, centrally acting:
Moxonidine

- **Lung disease**

drugs in the class mucolytics
Carbocisteine
1-Mucodyne Capsules
2-Carbocisteine capsules

- **Congestive heart failure**

Eplerenone

- **Renal disease**

Lanthanum
Fosrenal

- **Cancer**

Anastrozole
Tamoxifen
Bicalutamide
Oxaliplatin
Imatinib
Hydroxycarbamide
Capecitamine
Ruxolitnib

- **Ulcer disease**

*Put in a conditional argument (Include *azole or ranitidine if not taking NSAIDS or concomitant systemic glucocorticoid steroid.

Drugs in the class proton pump inhibitors:
Lansoprazole
Omeprazole
Pantoprazole
Rabeprazole sodium

Pariet gastro-resistant tablets [k4]
Rabeprazole gastro-resistant tablets [k4]
Ranitidine
Famotidine

- **Osteoporosis**

*Put in a conditional argument: If prednisolone AND Zoledronic acid Or Alendronic acid or Ibandronic acid, then do not code as osteoporosis

Zoledronic acid
Alendronic acid
Ibandronic acid
Raloxifene hydrochloride
Denosumab:
Denosumab 60/lml
Denosumab (Prolia)

- **Dementia**

Donepezil
Memantine

- **AIDS**

  Efavirenz
  Atripla

- **Anxiety & Panic Disorders**

  Diazepam
  Nitrazepam tablets
  Lorazepam

- **Depression**

  Depefex XL capsules
  Citalopram tablets
  Cipramil
  Imipramine
  Lithium
  Lofepramine
  Mirtazapine
  Phenelzine
  Reboxetin

- **Neurological**

  Dopamine precursors drugs:
  Co-careldopa
  Co-careldopa tablets
  Sinemet tablets
  Co-beneldopa (ingredients: levodopa and benserazide):
  Co-beneldopa capsules
  Madopar tablets
  Dopamine receptor agonists:
  Acute drug-induced dystonic reactions
  Repinex XL tablets
  Pramipexole modified-release tablets
  rotigotine patches
  Antimuscarinics drugs:
  Trihexyphenidyl hydrochloride
  Trihexyphenidyl 2mg tablets
  Procyclidine 5mg tablets
  Drugs in the class monoamine-oxidase b inhibitors
  Rasagiline
  Rasagiline tablets
  Azilect tablets
  Catechol-o-methyltransferase inhibitors class drugs
  Entacapone tablets
  Other:
  Ongentys hard capsules
  Stanek tablets (levodopa with carbidopa and entacapone)
  Amantadine hydrochloride tablets

- **Visual impairment**

  (Includes glaucoma irrespective of visual impairment)
  Drugs in the class antimuscarinics:
  Cyclopentolate hydrochloride (Mydrilate)
  Minims cyclopentolate hydrochloride eye drops
  Drugs in the class prostamides:
  Bimatoprost with timolol (eyzeetan, Ganfort)
  Bimatoprost eye drops (Lumigan, Eyreida)
  Drugs in the class prostaglandins and analogues:
  Latanoprost (Medizol, Monopost, Xalatan, Xelpros)
  Travoprost (Bondulc, Travatan, Izba)
  Timolol 5mg/ml eye drops
  Drugs in the class beta blocking agents, non-selective:
  Timolol maleate (azarga, cosopt, timoptic, betimol, tiopex)

- **No specific drug for the condition**

  - Congestive heart failure
  - Degenerative disc disease
  - Myocardial infarction
  - Cerebrovascular disease
  - Peripheral vascular disease
  - Liver disease
  - Hearing impairment
  - Hemiplegia
  - Diabetes end with end organ damage (requires additional comorbidities of retinopathy, neuropathy, or nephropathy to be present)

- **Additional Table 3. Morbidity count by checklist and augmentation (n=6,342)**

Morbidity	Painful Yes/No	Check-list [1] n	Free text [2] n	Medications [3] n	Total n, (%)
**Arthritis**	Yes	1447	21	9	1477 (68)
**Hypertension**	No	779	42	35	856 (39)
**Degenerative disc disease**	Yes	767	24	0	791 (36)
**Upper gastro-intestinal**	Yes	0	26	658	684 (31)
**Asthma**	No	325	7	81	413 (19)
**Diabetes without complications**	No	349	12	25	386 (18)
**Osteoporosis**	Yes	218	4	29	247 (11)
**Cancer**	Yes	201	30	6	237 (11)
**Angina**	Yes	197	14	17	228 (10)
**Lung disease**	No	139	38	8	185 (8)
**Myocardial Infarction**	Yes	150	0	0	150 (7)
**Stroke**	No	126	19	0	145 (7)
**Kidney disease**	No	116	10	0	124 (6)
**Depression**	Yes	0	23	81	104 (5)
**Visual impairment**	No	0	34	57	91 (4)
**Chronic Heart Failure**	Yes	60	2	1	63 (3)
**Neurological**	No	0	35	21	56 (3)
**Anxiety**	No	0	18	22	40 (2)
**Lower Gastro-intestinal**	Yes	0	29	0	29 (1)
**Hearing impairment**	No	0	12	0	12 (0.5)
**Diabetes with complications**	Yes	0	12	0	12 (0.5)
**Dementia**	No	9	0	0	9 (0.4)
**Liver disease**	Yes	0	7	0	7 (0.3)
**Peripheral vascular disease**	Yes	0	6	0	6 (0.3)
**Hemiplegia**	No	0	1	0	1 (<0.1)
**AIDS**	No	0	1	0	1 (<0.1)
**Subtotal illnesses in FRAIL (11)**		3,889	196	182	4,264
**Subtotal illnesses not in FRAIL (15)**		994	220	868	2,078
**Total morbidities identified**		4,883	413	1,050	6,342

Abbreviations: AIDS – Acquired immune deficiency syndrome. Items in bold are included in FRAIL, non-FRAIL illnesses are extra morbidities from Charlson and Functional Comorbidity Indices.
Diabetes is classified as painful if the participant reports complications such as neuropathy (Charcot foot), and retinopathy.
1 - morbidity appeared in checklist. This included all FRAIL illnesses plus degenerative disc disease, osteoporosis, and dementia. 2 - morbidity appeared in free text only. 3 - morbidity is inferred from medication only.

Note: there is an order of precedence to columns, the morbidity is counted as present in the following order 1) checklist; 2) free text; 3) medication; and counted once even though it may be originally reported in all three ways.
1159 (53%) of participants reported free text morbidities and 2088 (96%) reported medications.

- **Additional Table 4. Bivariate associations between morbidity count and covariables.**

Variable	*'Any' morbidities*	*Bivariate association β Coef. (95% CI)*	*Painful morbidities*	*Bivariate association β Coef. (95% CI)*	*Non-painful morbidities*	*Bivariate β Coef. (95% CI)*
Sex:						
Male,	3 (2-4)	Ref	2 (1-2)	Ref	1 (1-1)	Ref
Female	3 (2-4)	0.14 (003, 0.29), p=0.045	2 (1-3)	0.17 (0.07, 0.28), p=0.001	1 (0-2)	-0.03 (-0.11, 0.05), p=0.455
Age (years)	73 (69-79) *	0.01 (-0.001, 0.02), p=0.064	73 (69-79) *	0.01 (0.001, 0.02), p=0.02	73 (69-79) *	0.001 (-0.005, 0.007), p=0.746
BMI Classes#						
Underweight	3 (1-4)	0.17 (-0.44, 0.78), p=0.582	2 (1-3)	0.25 (-0.21, 0.71), p=0.289	1 (0-1)	-0.09 (-0.44, 0.26), p=0.616
Normal	2 (1-4)	Ref	2 (1-3)	Ref	1 (0-1)	Ref
Pre-obese	3 (2-4)	0.20 (0.03, 0.37), p=0.021	2 (1-3)	0.13 (-0.003, 0.26), p=0.055	1 (0-2)	0.07 (-0.03, 0.17), p=0.134
Obese	3 (2-4)	0.52 (0.34, 0.71), p<0.001	2 (1-3)	0.27 (0.13, 0.41), p<0.001	1 (1-2)	0.26 (0.15, 0.36), p<0.001

*Median age of participants with ≥1 morbidity count. Data are from n=2,155 participants
Abbreviations: BMI – Body Mass Index; β Coef. –beta coefficient; CI – 95% confidence intervals; Ref - reference group. #WHO classification for BMI (kg/m2), underweight <18.5, normal 18.5-24.9, pre-obese 25-29.9 and >30 obese.

- **Additional Table 5. Associations of frailty with standardised NRS pain and other characteristics.**

		**Frailty**
**Factor**	Interval/category	aOR (95%CI)
NRS pain	Standardised NRS pain	3.32 (2.79, 3.95), p<0.001
Painful comorbidities	Standardised count	1.37 (1.20, 1.56), p<0.001
Non-painful comorbidities	Standardised count	1.39 (1.24, 1.58), p<0.001
Sex	Male	Ref
	Female	1.36 (1.04, 1.76), p<0.024
Age	Years	1.03 (1.01, 1.05), p=0.001
BMI Class[#]	Underweight	3.49 (1.34, 9.09), p=0.011
	Normal	Ref
	Pre-obese	1.43 (1.01, 2.01), p=0.041
	Obese	2.15 (1.53, 3.02), p<0.001
Pseudo r^2		0.2323

The outcome measure is binary frailty without morbidity (mFRAIL), defined as mFRAIL score >2. The model is frailty with standardised painful and non-painful comorbidities adjusted for standardised pain severity, age, sex, and BMI class. Data are from n=2,015 participants. Standardized coefficients represent the change in the dependent variable's standard deviation associated with a one-standard-deviation increase in the predictor variable, they permit comparison of variable with different scales. Abbreviations: NRS – numerical rating score; BMI – Body Mass Index; aOR –adjusted odds ratio; CI – 95% confidence intervals; Ref - reference group. NA - Not Applicable [#]WHO classification for BMI (kg/m2), underweight <18.5, normal 18.5-24.9, pre-obese 25-29.9 and >30 obese.
